# Postoperative chylothorax following video-assisted mediastinoscopic lymphadenectomy (VAMLA) for early-stage non-small cell lung carcinoma

**DOI:** 10.1093/jscr/rjae665

**Published:** 2024-10-28

**Authors:** Kayleigh A M van Dam, Yvonne L J Vissers, Karel W E Hulsewé, Erik R de Loos

**Affiliations:** Department of Surgery, Division of General Thoracic Surgery, Zuyderland Medical Center, Henri Dunantstraat 5, 6419 PC, Sittard-Geleen and Heerlen, The Netherlands; Department of Surgery, Division of General Thoracic Surgery, Zuyderland Medical Center, Henri Dunantstraat 5, 6419 PC, Sittard-Geleen and Heerlen, The Netherlands; Department of Surgery, Division of General Thoracic Surgery, Zuyderland Medical Center, Henri Dunantstraat 5, 6419 PC, Sittard-Geleen and Heerlen, The Netherlands; Department of Surgery, Division of General Thoracic Surgery, Zuyderland Medical Center, Henri Dunantstraat 5, 6419 PC, Sittard-Geleen and Heerlen, The Netherlands

**Keywords:** chylothorax, uniportal video assisted thoracoscopic surgery, video-assisted mediastinoscopic lymphadenectomy, thoracic surgery

## Abstract

Chylothorax is a rare condition in which chylous fluid accumulates in the pleural cavity, most often due to iatrogenic injury of the thoracic duct. This paper reports a case of a woman in her 50s presenting with chyle leakage after a video-assisted mediastinoscopic lymphadenectomy (VAMLA) for staging of a nodule suspected of non-small cell lung carcinoma. After the VAMLA, a Uniportal Video Assisted Thoracoscopic Surgery lobectomy was scheduled during which the chylothorax was observed. The chyle was evacuated and the planned resection was conducted. The chest drain could be removed after 6 days while the patient followed a medium-chain triglyceride diet for 4 weeks. The purpose of this case report is to raise awareness for this very rare complication after VAMLA and discuss treatment options.

## Introduction

Chylothorax is the accumulation of chylous fluid in the pleural cavity, most often due to iatrogenic injury of the thoracic duct. After intrathoracic procedures a chylothorax is seen in up to 0.5% of all cases and is most frequently observed after esophagectomy (5%–10%) and lung resection with mediastinal lymph node dissection (3%–7%) [1, 2]. Occurrence of chylothorax following Video-Assisted Mediastinoscopic lymphadenectomy (VAMLA) is very rare. In the largest published case series, no chylothorax was reported after 600 VAMLA’s [3]. In general, the surgical complications are bleeding and infection. In addition to these complications there are other risks associated with the VAMLA procedure. These risks can be intraoperative, including iatrogenic injury to the trachea, bronchus, vena cava, azygos vein, esophagus, pulmonary artery, recurrent nerve, and parietal pleura. The postoperative complications can be hemorrhage and mediastinitis as mentioned in the article by Lozekoot *et al.* [4].

Postoperative chylothorax after pulmonary surgery may be caused by damage of the branches of the thoracic duct during mediastinal lymph node dissection [5, 6]. The highest rate is reported following surgery of the right lung, possibly because of the greater volume of chyle draining from the left pleural space to the right space [6, 7]. Current literature suggests that histology and lung cancer stage do not influence the occurrence rate [8].

The sequelae of chylothorax vary in severity and include malnutrition and immune dysfunction. Especially with continuous leakage and the loss of fluids and lymphocytes may lead to the before mentioned consequences [9]. When left untreated, mortality rate is high and ranges from 30% to 50% [9–11]. Possible contributors to the upper range of the high mortality rate are patients with a bilateral or malignant chylothorax [9–11]. The treatment of a chylothorax comprises of non-operative and surgical options. Conservative therapy consists of fasting, total parenteral nutrition (TPN) or following a medium-chain triglyceride (MCT) diet [12]. The latter is preferred since MCT are not absorbed by intestinal lymphatics and therefore potentially reduce chylous flow [12]. Even though a systematic review showed a slightly higher success rate with MCT diet compared to TPN, there were no statistically significant differences reported [13].

There are studies that suggest that chyle drainage of >1000 ml per 24 hours (high-output) is an indication for (re)intervention [14–16]. Possible treatment options are radiological thoracic duct embolization (TDE) and surgical duct ligation (SDL) [14]. We present a case of chylothorax after VAMLA which was successfully treated with chest tube drainage and a MCT diet.

## Case report

A female patient in her 50s with a body mass index (BMI) of 24.3 kg/m^2^ was evaluated for metabolically active ground glass opacification of 48 mm, partially solid, in the lower lobe of the right lung. The lesion was -positron emission tomography (PET) positive and detected during routine follow-up of a urothelial carcinoma for which she had undergone a transurethral resection previously. In multidisciplinary team discussion the nodule was suspected to be a non-small cell lung carcinoma (NSCLC) and was staged as cT2bN0M0 according to the TNM classification (8^th^ edition). To rule out mediastinal lymph node metastasis, surgical mediastinal staging via VAMLA was performed. As N2-disease was ruled out, surgical treatment was proceeded by a minimally invasive lobectomy via Uniportal Video Assisted Thoracoscopic Surgery (VATS).

The VAMLA was performed according to the applicable standards, during which a complete mediastinal lymphadenectomy of the paratracheal, parabronchial and subcarinal lymph node stations was carried out. The lymph node dissection was performed en-bloc, with surrounding fat tissue, as per standard protocol for VAMLA. There were no perioperative complications such as tearing of the lymph nodes, bleeding, or the presence of chyle-like fluid. The early postoperative course was uneventful, and the patient could be discharged from the hospital the same day.

### Investigations and treatment

Four days after the VAMLA the patient presented at the emergency department with dyspnea and hoarseness. A modified Early Warning Score of 1 due to a heart rate of 102 beats per minutes was observed. Furthermore, a temperature of 37.7 degrees Celsius, a blood pressure of 162/104 mmHg and an oxygen saturation of 98% without additional support were registered. Laboratory tests showed a white blood cell (WBC) count of 7.8 x 10*9/L (normal 4–10) and a slightly elevated C-Reactive Protein (CRP) of 43 mg/L (normal <10) which was attributed to postoperative healing.

**Figure 1:**
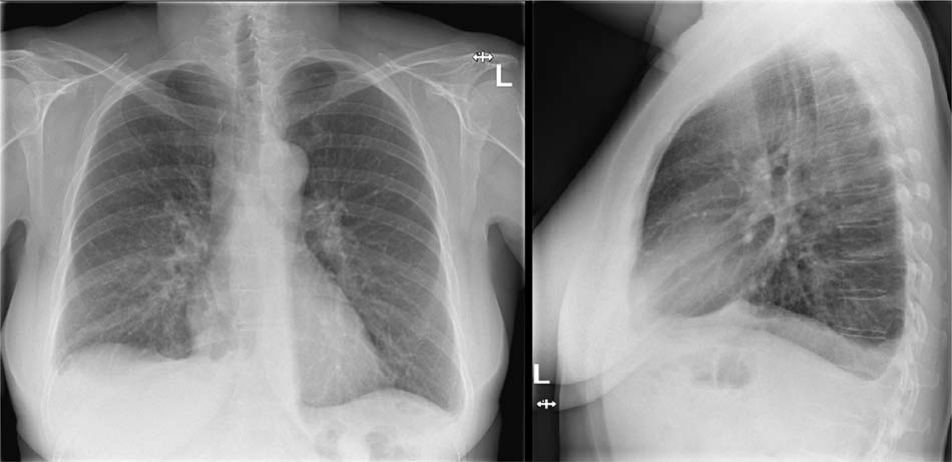
Chest X-ray during visit to the emergency department showing bilateral pleural effusion.

**Figure 2:**
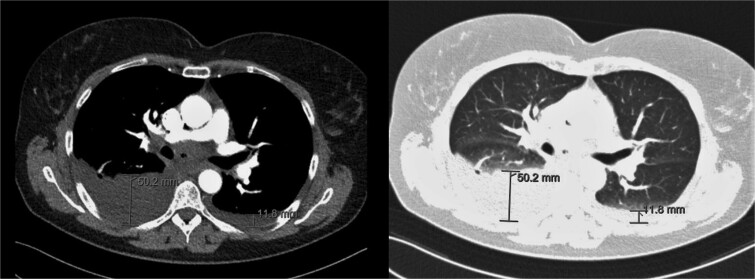
CT-scan during visit to the emergency department confirming the pleural effusion attributed as reactive or postoperative.

Additional imaging diagnostics with X-ray demonstrated bilateral pleural effusion, more apparent at the right side (Fig. 1). A subsequent computed tomography (CT) scan showed no signs of pulmonary embolism and confirmed the pleural effusion (Fig. 2). In addition, air was seen in the anterior mediastinum (pneumomediastinum). Both air and pleural effusion were attributed to normal healing after the recent VAMLA, and in the absence of clinical alarm symptoms the patient was sent home. In the absence of loculation or contrast captation, the bilateral pleural effusion had no radiological signs of empyema. One of the symptoms of pleural effusion is dyspnea which was also present in the patient. In addition, there was no suspicion of an infectious cause with normal vital values with a temperature of 37.7°C and no significant elevation of WBC or CRP. It was suspected that the effusion was postoperative or reactive to the suspected lung cancer. Regarding the hoarseness, the most likely cause was post-operative swelling of the surgical area. As the hoarseness did not occur immediately post-operative, iatrogenic recurrent nerve injury was not suspected.

**Figure 3:**
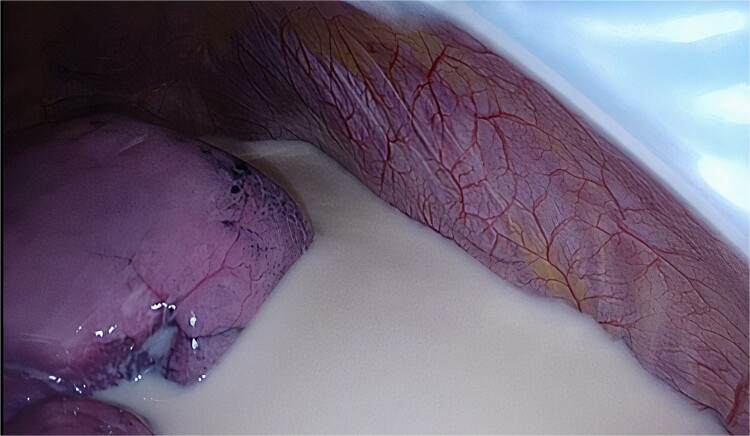
Intra-operative view of chyle.

The planned Uniportal VATS was performed under general anesthesia with 2 g of Cefazolin intravenously as antibiotic prophylaxis. Entrance to the right chest was obtained by an incision at the level of the fifth intercostal space in the midaxillary line. Upon inspection of the pleural cavity the presence of a milky fluid was noted, most likely in relation to chyle leakage after the previous VAMLA (Fig. 3). The fluid was collected and sent for cultivation and blood chemistry tests such as triglycerides. The parietal pleura appeared intact. After opening of the hilar pleura, the mediastinal tissue appeared infiltrated, possibly due to the chyle leakage. The hilar structures to the right lower lobe were dissected, after which the pulmonary arterial branches, upper lobe bronchus, and superior pulmonary vein were stapled, respectively. Finally, the fissure was completed, and the lower lobe was resected. A 28Fr chest tube was placed, and the lung was reinsufflated resulting in full expansion of the lung without signs of air leakage. The wound was closed in layers and the chest tube was connected to an underwater seal device.

### Follow-up

The patient was admitted to the thoracic surgical ward postoperatively. The cultures showed no aerobic or anaerobic growth. The chemistry tests did reveal triglycerides value in the fluid of >50 mmol/L, surpassing the diagnostic threshold for chyle of 1.24 mmol/L reported in the literature [17]. Because of the intraoperative observation of milky fluid a chyle leak was suspected and a MCT diet was started. In the next days, the chest tube produced only sero-sanguinous fluid while no more chyle was observed. With an initial production of 400 cc per day the tube could be removed after 6 days. The complaints of hoarseness and dyspnea were no longer present after Day 3 without the need for intervention. After removal of the chest tube a urinary tract infection was diagnosed which caused a delay in discharge of the patient until Day 10 postoperatively.

The examination of the resected lobe by the pathologist confirmed a well/moderately differentiated papillary adenocarcinoma. Multidisciplinary team-meeting concluded a pT3N0M0 NSCLC, stage IIB according to the TNM-classification (8^th^ edition). The upstaging from T2b to T3 in the postoperative diagnosis was due to a discrepancy between the tumor size measured on the preoperative CT scan and the final histopathological assessment. The preoperative imaging provides an estimate of the tumor size, but the definitive pathological examination offered a more accurate measurement whereby the tumor exceeded the 5 cm threshold. Adjuvant chemotherapy with carboplatin and pemetrexed was started 1 month postoperatively. During the follow-up after 3 months and 9 months postoperatively the CT-scans showed no indications of recurrence or metastases. Furthermore, no pleural effusion was noticed.

## Discussion

In general, chyle leakage with associated chylothorax is a rare complication that occurs in 0.5% of all intrathoracic procedures [1, 2]. Due to this low prevalence the number of reports in the literature are limited.

The literature is consistent with conservative treatment with chest drainage and diet-modifications being the first choice for chylothorax following different surgical procedures [1, 18, 19]. This non-operative treatment always consists of chest drainage and diet-modifications [1, 18, 19]. When this approach fails, e.g. in case of chyle leakage of >500–1000 ml per 24 hours, surgical intervention is warranted [14–16, 20, 21]. It is hypothesized that conservative treatment is less effective in cases with high flow. The patient in this report most likely had a chylothorax due to leakage of the side branches with a lower flow, thus resulting in a higher chance of successful conservative treatment. Consistent leakage after >5 days is also considered as an indication for intervention [1, 22].

Possible treatment options are thoracic duct embolization (TDE) and surgical duct ligation (SDL) [14]. If the chyle leak can be identified after surgical exploration, often thoracoscopically, SDL can be performed by direct ligation with a nonabsorbable suture or clip [14, 23]. The least invasive method however is TDE as it is a percutaneous procedure. During the TDE lymphangiography is performed and the cisterna chyli can be embolized [14]. Both coils and glue can be used as an embolic agent. However, if possible non-operative treatment with dietary measures is the first option of choice.

The patient presented in this report developed a chylothorax following mediastinal surgery, which was not recognized initially. Even though rare, chylothorax is a possible complication after VAMLA and is associated with damage to the thoracic duct branches. In this report the chyle leakage is presumed to have been caused by excessive accumulation of lymphatic fluid due to the mediastinal lymph node dissection during VAMLA, potentially due to blunt dissection. There was no evidence of iatrogenic injury of the thoracic duct. In addition, the mediastinal parietal pleura remained intact during the procedure; however, due to a substantial postoperative accumulation of chyle in the extrapleural mediastinal space the pressure increased leading to a subsequent defect in the parietal pleura. This would account for the presence of chyle in the pleural cavity despite no visible intraoperative breach in the pleura. In addition, this highlights the potential for delayed pleural involvement in cases of chylothorax. The first treatment of choice for chylothorax remains conservative as it is safe and effective and minimizes risks before considering surgery. The patient was treated with a MCT diet and chest drainage, emphasizing the significance of chest drains in managing chylothorax postoperatively as it allows evaluation of chylous fluid guiding possible further interventions.
